# Resilience and Burnout Among Healthcare Staff During COVID-19: Lessons for Pandemic Preparedness

**DOI:** 10.3390/healthcare14020195

**Published:** 2026-01-13

**Authors:** Daniela Bellicoso, Teresa J. Valenzano, Cecilia Santiago, Donna Romano, Sonya Canzian, Jane Topolovec-Vranic

**Affiliations:** 1 Professional Practice, Unity Health Toronto, Toronto, ON M5B 1W8, Canada; teresa.valenzano@unityhealth.to (T.J.V.);; 2 Li Ka Shing Knowledge Institute, St. Michael’s Hospital, Unity Health Toronto, Toronto, ON M5B 1X3, Canada; 3 Department of Speech-Language Pathology, Temerty Faculty of Medicine, University of Toronto, Toronto, ON M5G 1V7, Canada; 4 Lawrence S. Bloomberg Faculty of Nursing, University of Toronto, Toronto, ON M5T 1P8, Canada; 5 People, Mission, Culture, Unity Health Toronto, Toronto, ON M5B 1W8, Canada; 6 Institute of Health Policy, Management, and Education, University of Toronto, Toronto, ON M5T 3M6, Canada

**Keywords:** resilience, burnout, healthcare, mental health, pandemic preparedness, employee wellbeing

## Abstract

**Background/Objectives**: Healthcare workers at the frontline of managing pandemics are at increased risk for adverse physical and mental health outcomes, which has been shown to result in burnout. The relationship between personal resilience and burnout among clinical and non-clinical healthcare staff working in an acute care setting was assessed at the start of the COVID-19 pandemic. **Methods**: A prospective cross-sectional survey design with electronic questionnaires was used to measure resilience (Connor-Davidson Resilience Scale,) and burnout (Maslach Burnout Inventory—Human Services Survey). Linear regression analyses were conducted to examine the relationship between resilience and emotional exhaustion, depersonalization, and personal accomplishment. **Results**: A significant inverse relationship between resilience and both emotional exhaustion and depersonalization, and a positive relationship between resilience and personal accomplishment were identified. Higher resilience scores were significantly associated with lower emotional exhaustion and depersonalization and higher personal accomplishment under pandemic conditions. **Conclusions**: Strategies to boost resilience organization-wide amongst healthcare staff providing patient care are critical for providing skills to reduce the onset of burnout and support employee mental health. From a pandemic preparedness lens, organizational-level emergency management should consider the importance of resilience-building among staff to proactively prevent burnout and its subsequent effects on patient-care and general hospital functioning.

## 1. Introduction

Since 2003, the World Health Organization has declared several new epidemics and pandemics, including SARS, H1N1, MERS, Ebola, and most recently, the novel Coronavirus infectious disease (COVID-19). In addition to the health, social, economic, and political ramifications of pandemics, they place significant strain on healthcare staff and systems. Healthcare staff at the frontline of managing pandemics are at increased risk for psychological distress and burnout [1]. Specifically, poor mental health reduces productivity and quality of work output due to absenteeism and presenteeism among healthcare providers [2]. From an organizational management perspective, staff mental health and wellbeing are critical for supporting a hospital’s functioning and ability to provide patient care. As such, attempts to mitigate burnout among hospital staff should be considered under everyday- and pandemic-conditions to diminish its downstream effects on staff wellbeing, organizational functioning, and patient care.

Various factors (e.g., age, gender, sleep, career stage, chronotype, personality, work shift) have been linked healthcare provider burnout [3,4,5,6,7], but these factors are not always easy or possible to change. Resilience, however, is a modifiable factor known to impact burnout amongst healthcare providers [8,9]. To date, much of the research on the relationship between resilience and burnout has focused on specific groups of healthcare providers and/or on staff working in specific units. While this literature is relevant at the discipline-specific or unit level, it does not examine the association from an organizational or system-level lens, creating a paucity of literature in this area. We took an organization-wide approach and examined various healthcare staff providing patient care across our hospital to understand the importance of resilience across an organization’s staff as a preventative tool against burnout under pandemic settings. As the pandemic introduced unprecedented occupational demands, pressures, and moral distress across healthcare systems, this raised important questions about the limits of resilience as a protective factor. Understanding whether resilience continues to mitigate burnout under such conditions across the staff within a healthcare organization is critical for informing workforce wellbeing strategies and organizational interventions.

### Background

Burnout often occurs among individuals who engage in ‘people-work’ such as caring—or providing service—for others, and includes feelings of emotional exhaustion (EE, sense of being emotionally overextended and/or exhausted by one’s work), depersonalization (DP, feelings of being detached, unsympathetic, or providing an impersonal response towards patients in one’s care), and lack of personal accomplishment (PA, feelings of proficiency and accomplishment in one’s work with people) [10]. In addition, burnout is known to reduce quality of patient care and satisfaction [11,12], decrease workplace performance (i.e., elevated presenteeism), and exacerbate the need for sick leave [2], subsequently increasing costs for the employee’s organization.

Resilience, a suggested protective factor against burnout, refers to one’s ability or personal qualities that allow them to cope well in the face of adversity and deal with the stress of a situation [13]. The theoretical understanding of resilience has evolved over the decades. Until recently, resilience has generally been conceptualized as involving exposure to significant adversity (from ongoing daily hassles to major life events), and the display of positive adaptation [14,15]. It has been examined from various perspectives, including from developmental and trait-based lenses, as a dynamic and evolving process that varies across time and situations, and as having neurobiological ties. More recently a cohesive model has been proposed that synthesizes cognitive, emotional, social, and physiological mechanisms into a unified, actionable structure [14]. Resilience can help to improve one’s defence against burnout by equipping the individual with tools to effectively cope with the stress of a particular situation [8,16,17,18]. Strategies that have been employed to effectively build or sustain resilience amongst healthcare workers include resilience training workshops and e-learning modules, availability of support and wellbeing measures, and professional acknowledgment [19,20,21,22].

While resilience has been shown to reduce burnout under typical (i.e., non-pandemic) work conditions in healthcare (e.g., [8,9,20]), research is needed to understand its protective benefit under pandemic situations when people are faced with increased demands and heightened levels of workplace stress. Furthermore, while much work has focused specifically on the relationship between burnout and resilience among doctors or nurses, more research is needed that also examines the relationship between resilience and burnout amongst healthcare staff (including, but not limited to nurses, health disciplines staff, clericals, etc.) working to provide patient care.

The objective of this study was to examine the potential protective association between resilience and burnout among healthcare staff during the start of the COVID-19 pandemic in 2020 with the intention of supporting pandemic preparation and organizational emergency management. The authors hypothesized that higher resilience would be associated with reduced feelings of burnout (i.e., lower EE and DP, and higher PA).

## 2. Materials and Methods

### 2.1. Study Design

This research applied a prospective cross-sectional design to examine the association between resilience and self-reported burnout among frontline healthcare staff during the initial wave of COVID-19 at an urban inner city health sciences centre in Toronto, ON, Canada.

### 2.2. Settings

This study was conducted at a large, urban, inner-city academic health sciences centre in Toronto, Ontario, Canada. The hospital includes 519 inpatient beds, provides tertiary and quaternay care, and houses a Level-1 Adult Trauma Centre. The results of this paper examine data collected online between 3 May and 3 July 2020. Data collection took place online via SurveyMonkey. Participants accessed the study questionnaire via an electronic link and were able to complete the survey either onsite at the hospital or offsite. This study received ethics approval from the institutional Research Ethics Board (Protocol #20-085).

### 2.3. Participants

Eligible participants included clinical (nurses, health disciplines, and physicians) and non-clinical healthcare staff working at the study organization for a minimum of two or more days per week. Participants were included if they were interacting with and/or providing care to patients under investigation (PUIs) or patients with COVID-19. Participants were recruited via various internal channels (e.g., email recruitment messages on behalf of the study team, announced the study in meetings, and highlighted the study in two separate electronic hospital newsletters).

### 2.4. Data Collection

Data collected included a demographic questionnaire to describe the sample, and validated measures of resilience and burnout. Resilience was measured using the 25-item Connor-Davidson Resilience Scale (CD-RISC25) [13], one of the most widely used measures of resilience in research and practice. The CD-RISC25 includes 25 questions, rated on a 5-point Likert scale (0, not true at all, to 4, true nearly all the time) summed to yield a total score from 0 to 100 (100 indicates high resilience). Burnout was measured using the Maslach Burnout Inventory—Human Services Survey (MBI-HSS) [10,23], the gold standard for measuring burnout. The MBI includes 22 questions rated on a 7-point Likert scale (e.g., “I feel burned out from my work”: 0—never, 1—a few times a year or less, 2—once a month or less, 3—a few times a month, 4—once a week, 5—a few times a week, 6—everyday). This scale produces three separate subscales (EE, DP, and PA) whose scores range from 0 (low) to 6 (high) for the particular trait when calculated using the “average” scoring method [23]. All participants completed the same instruments using identical assessment methods via online survey. To minimize selection bias, recruitment materials were broadly distributed across the organization, and targeted clinical and non-clinical patient-facing staff. Standardized, validated self-reported measures reduced the risk of measurement bias, however given the voluntary nature of participation and reliance on self-reported data, the potential for response bias (including self-selection, and social desirability bias) are acknowledged. In an effort to mitigate bias and obtain responses from a wide range of participants, calls for participation were circulated through various channels including hospital newsletters for employees, email blasts, and calls from unit managers. Convenience sampling was applied in this study, given the constraints that many healthcare staff were working under at the time; the study team did not want to add additional undue pressure on staff who may have been unable to commit the time necessary to participate given the pandemic situation and its associated strains on the healthcare system.

### 2.5. Quantitative Variables

Resilience and burnout (subscale) scores were treated as continuous variables in all analyses. No categorization or grouping of these variables was performed. While both these questionnaires comprise Likert-type items, these tools are validated multi-item scales with wide score ranges. The composite scores of these measures approximate continuous distributions.

### 2.6. Statistical Methods

All statistical analyses were completed using SPSS version 24.0 (Armonk, NY, USA) [24]. Descriptive statistics were calculated for all of the demographic variables: means and standard deviations for continuous data, and frequency counts and proportions for categorical data. Linear regression was used to evaluate the relationship between resilience, as measured by the CD-RISC25, and the three components of burnout as measured by the MBI (EE, DP, and PA). Demographic variables were not included as covariates in the regression analyses, as the study aimed to examine the overall association between resilience and burnout across patient-facing clinical and non-clinical staff during the early phase of the pandemic, rather than to model the independent effects of individual demographic or occupational factors.

## 3. Results

In total, 275 participants completed this baseline data collection phase of the study, including completion of the demographic survey, CD-RISC25, and the MBI. All analyses were conducted with the data from the 275 survey respondents. See Table 1 for a summary of the cohort demographics. Over 80% of respondents were female. Fifty-five percent of this cohort were between the ages of 20 and 39. Eighty-five percent of the participants reported having completed at least one post-secondary degree, and 80% reported completing a graduate degree. Of all the respondents, over 92% indicated they were in a clinical role (i.e., physician, nurse, health discipline clinician). Nurses and health discipline clinicians (e.g., social worker, registered dietitian, or respiratory therapist) comprised the majority of respondents (88.8%).

For resilience, on a summed scale of zero (0) to one hundred (100) (higher scores represented increased resilience), the mean score was 70.96 (SD = 12.145). For the MBI, on a scale from zero (0, *never feel burned out from my work*) to six (6, *feel burned out from my work every day*), where a higher score represented greater feelings of the particular subscale component, across all participants, the mean component scores were EE: 3.01 (SD = 1.298), DP: 1.53 (SD = 1.275), and PA: 4.60 (SD = 0.819). These burnout scores are computed according to the “average” scoring method as outlined in the MBI Manual [23]. (N.B., in the literature, some studies present the scores of the MBI using the “sum” scoring method [23]. For comparative purposes, the “sum” scores for each component of the MBI scale were EE: 27.09, DP: 7.65, and PA: 36.80.)

### 3.1. Relationship Between Resilience and Emotional Exhaustion

**Figure 1:**
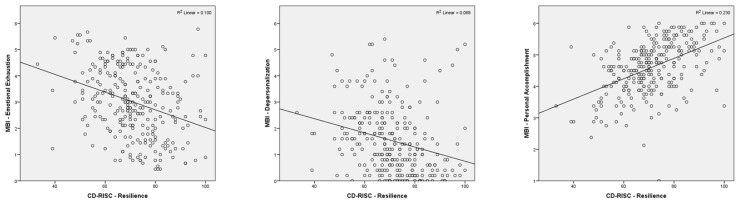
Regression plots demonstrating the relationship between the independent variable (resilience) and dependent variables (burnout components—EE, DP, and PA), and the corresponding proportion of variance in the dependent variable accounted for by the independent variable (R^2^).

Linear regression was conducted to explore the relationship between scores on the EE subscale of the MBI and resilience scores as reported on the CD-RISC25 in healthcare workers. A significant relationship was found between EE and resilience (F(1, 273) = 30.212, *p* < 0.001), establishing that a healthcare worker’s reported CD-RISC25 resilience score could statistically significantly predict their score on the EE subscale of the MBI, using the following regression equation: predicted EE subscale on the MBI = 5.403 + [−0.034 × (resilience score on the CD-RISC25)]. The resilience score provided by the CD-RISC25 accounted for 10.0% of the explained variability in the MBI EE subscale score. For each single point increase in resilience score, scores on the EE subscale decreased by 0.034 (95% CI, −0.046 to −0.022) points (Figure 1).

### 3.2. Relationship Between Resilience and Depersonalization

A linear regression was conducted to explore the relationship between resilience scores on the CD-RISC25 on the DP subscale of the MBI. This revealed a statistically significant relationship between resilience and DP (F(1, 273) = 19.978, *p* < 0.001), with resilience scores accounting for 6.8% of the variation in scores on the DP subscale of the MBI. The prediction equation was as follows: score on the DP subscale of the MBI = 3.447 + [−0.027 × (resilience score on the CD-RISC25)]. This indicates that for each single point increase in resilience as measured by the CD-RISC25, scores on the DP subscale of the MBI decreased by 0.027 (95% CI, −0.039 to −0.015) points (Figure 1).

### 3.3. Relationship Between Resilience and Personal Accomplishment

A linear regression evaluating the relationship between reported resilience scores on the CD-RISC25 and the PA subscale of the MBI revealed a statistically significant relationship (F(1, 273) = 81.647, *p* < 0.001), with resilience scores accounting for 23.0% of the variation in scores on the PA subscale of the MBI. The prediction equation was as follows: score on the PA subscale of the MBI = 2.302 + [0.032 × (resilience score on the CD-RISC25)]. This relationship suggests that for each single point increase in score on the CD-RISC25 results in a 0.032 score increase on the PA subscale of the MBI (95% CI, 0.025 to 0.039) (Figure 1).

## 4. Discussion

Our results indicated that at the start of the COVID-19 pandemic, healthcare staff at a large urban inner-city hospital with elevated resilience reported lower burnout. Higher resilience scores predicted diminished EE [0.034 (95% CI, -0.046 to -0.022)] and DP [0.027 (95% CI, −0.039 to −0.015)], and an increased sense of PA [0.032 (95% CI, 0.025 to 0.039)]. Similar results have been found during non-pandemic times (e.g., [25,26]) and during the early months of the pandemic [27,28], supporting the value of regular resilience building and maintenance activities among healthcare staff to reduce feelings of EE and DP, and improve one’s sense of PA. This information can be used to support the prevention of mental health problems in the workplace across disciplines. This work aligns with evidence that boosting healthcare staff resilience is an important and achievable way to protect against burnout especially during the onset of a pandemic (e.g., [8,9,27]), and supports the implementation of resilience-building initiatives across healthcare staff and hospital units. To date, findings have been mixed on whether resilience impacts all three aspects of burnout (EE, DP, and PA [27,29], or only certain components (e.g., [30]). Our findings support the association between resilience and each component of burnout during the early months of a pandemic under rapidly changing conditions. Much of the research has focused on the relationship between resilience and burnout in specific groups of healthcare staff and/or among groups working in a specific care unit. Our study builds on this work by examining the impact of resilience on burnout among staff (i.e., health disciplines staff, nurses, physicians, and clericals) providing patient care in the early phase of the COVID-19 pandemic, across various care units within an inner city academic acute care health sciences centre; the findings indicate that even in this high-stress period where guidelines and work conditions were constantly changing, resilience can lower negative feelings of EE and DP, and improve the positive feeling of PA. These results, coupled with previous research indicating that diminished burnout reduces absenteeism and presenteeism among healthcare staff [2,31] can be used to support mental health in the workplace and show that even under pandemic settings, employee mental health can be protected.

Resilience scores reported in our study (70.96 (SD = 12.145.729) were comparable to scores reported in the CD-RISC manual (data collected pre-COVID-19 [32]) and in recent studies (e.g., amongst resident physicians and nurses, 68.5 and 66.5, respectively [33,34]), including those relating to the COVID-19 pandemic with mean scores ranging between 60 to mid-70 [28,35,36,37,38,39,40]. Participants reported robust resilience levels, suggesting they were equipped with the necessary skills to overcome some degree of stress and or adversity in their daily lives.

Burnout subscale scores reported in our study were EE: 3.01 (SD = 1.298), DP: 1.53 (SD = 1.275), and PA: 4.60 (SD = 0.819). Burnout scores as measured by the MBI do not have a definitive cut-off score that proves one is (or is not) burned out [23]. Compared to MBI-HSS norms for individuals working in “medicine” (based on data collected prior to the COVID-19 pandemic) [23], our participants study reported higher EE (i.e., a few times a month, based on the MBI scoring scale). Respondents reported similar levels of DP (i.e., more than a few times a year, but less than once a month) and PA (i.e., more than once a week) to the published MBI-HSS norms. As we do not have data on our participants’ burnout levels prior to the COVID-19 pandemic, we cannot comment on whether these data reflect a difference in how participants felt pre-pandemic versus during the early months of the pandemic. However, a systematic review of the nursing burnout literature between 1994 and 2022 [41], reported no significant differences in quantitative scores from prior to, compared to during the COVID-19 pandemic for EE, DP, and PA, yet in their qualitative assessments, they noted that participants reported heightened EE and reductions in PA during the COVID-19 period [41]. (N.B., qualitative data were collected from our study participants, however for the scope of this paper, the data were not included.) Compared to the findings of other early pandemic studies [27,28,42], our results are similar to the overall general trend indicating that participants experienced some EE and DP, but experienced a greater level of PA. This suggests that amongst our participants, even with some degree of EE and DP, a sense of PA was experienced.

The gender distribution of the sample, which included a higher proportion of women, is consistent with the composition of the healthcare workforce. Research demonstrates that over 70% of health human resources are women [43]. In our study, nurses accounted for a substantial proportion of the study, and nursing as a field is known to be predominantly female [44] which can contribute to the explanation of the study having a higher proportion of women. In addition, research has demonstrated that while women are less likely to partake in clinical trials compared to men, females are typically significantly more willing to partake in survey-based research [45,46] such as that used our study. In light of these details, the observed gender distribution likely represents underlying workforce and engagement patterns rather than systematic overrepresentation.

While various studies have examined the connection between resilience and burnout during COVID-19 among specific groups of healthcare providers and or on specific units (e.g., [28,30,47,48]) from a strengths perspective, we took an organization-wide approach and examined healthcare staff across our urban, academic health sciences hospital instead of only examining specific groups. Our findings support the need for organizations as a whole to work towards improving staff resilience, especially under pandemic conditions, to reduce burnout.

Another strength of this work was our use of validated tools. The MBI is considered the gold standard for measuring burnout [49,50] and corresponds to the World Health Organization’s conceptualization of burnout [51]. While no “gold standard” appears to exist in the literature for measuring resilience, the CD-RISC is a widely accepted, validated measure. Both these tools offer good reliability and validity, and have published norms against which we were able to compare our data.

In addition, this study employed real-time, prospective data collection, rather than retrospective accounts. Given the known negative impact of stress on memory consolidation and retrieval [52,53,54] it is possible that one’s memory of their burnout and resilience may not have been accurate if reported retrospectively following those early, uncertain months of the pandemic. At the start of the pandemic, many clinical and non-clinical staff were redeployed to new roles to meet pandemic needs. Many staff who may not have previously been patient-facing were now working in some capacity in a patient-facing role and as such were included within our study. This allowed us to include participants who are not typically considered as being at potential risk of burnout and or in need of resilience training on account of interacting with patients (i.e., all study participants were patient-facing). The inclusion of these participants within the study population indicates that as part of pandemic preparedness within a healthcare organization, resilience training for burnout prevention should be offered to all staff. Our findings highlight the need for all staff to have a strong sense of resilience, in order to be prepared for changes or shifts within a hospital organization that may potentially increase one’s burnout level. Beyond pandemic situations, amongst staff who are patient-facing, resilience can be a protective factor that potentially reduces burnout and helps people to stay in their role. This could contribute to reducing the number of staff who leave their roles due to feelings of burnout, which ultimately affects staffing levels and patient care. Within our own organization, after these data were collected, various initiatives were implemented to build resilience (e.g., resilience-training workshop [19]) and support employee wellness and joy at work. These initiatives are intended to build one’s resilience and protect against burnout by helping people develop skills to cope well and see benefit in the work they are carrying out in the face of adverse and/or stressful situations. The initiatives also provided staff with strategies to care for themselves and foster healthy work environments, improved health system outcomes, and enhanced patient care. Additional strategies that have been found to build resilience among healthcare staff include adhering to positive thinking, maintaining individual and/or social-organizational support, practicing cognitive problem-solving skills, and building emotional intelligence [55,56].

While this study identified several relevant findings, it is important to acknowledge some of its limitations. While the study site is a large organization (519 inpatient beds, level 1 trauma centre, provides tertiary and quaternary care), data were collected from a single site, and represent the experiences of respondents from one organization. Our data were collected over eight-weeks (May to July 2020) when our organization (along with hospitals worldwide) was rapidly adjusting and updating their guidelines to adapt to the changing landscape and patient needs, and might therefore reflect a more heterogeneous participant experience. In addition, the use of human subjects introduces the potential for selection bias (i.e., at this time, the potential exists that participants with particularly high burnout may have opted not to partake). Furthermore, as all measures were self-reported, the possibility of reporting biases (e.g., social desirability, recall bias) may have influenced participants’ responses at the time of reporting. Nonetheless, our findings do align with other larger multi-site studies that support the need for resilience building to mitigate the effects of burnout among healthcare providers [57,58,59]. Considering the study’s limitations and strengths, overall we feel this work presents valuable information highlighting the importance of building resilience across a healthcare organization’s staff, particularly during pandemic times, to reduce EE and DP, increase PA, and protect employees’ mental wellbeing.

### Strengths, Limitations & Future Directions

Future resilience and burnout research under pandemic conditions should examine the influence of these constructs on patients’ satisfaction with their care to advance the patient-satisfaction literature. From a practice standpoint, these results support continued resilience building efforts amongst interprofessional staff within healthcare organizations, not only for pandemic preparedness, but also for preventing feelings of burnout that occur in non-pandemic settings. Within our institution, following the collection of this study data, an interprofessional organization-wide resilience building workshop was offered [19] with the goal of providing staff with strategies to care for themselves and foster healthy work environments, improved health system outcomes, and enhanced patient care. Going forward, interprofessionally based resilience building initiatives can be taken both organization-wide and within hospital units, in order to bring various staff together to collectively build resilience and support one another.

While research has previously demonstrated that patients cared for by staff experiencing burnout report lower satisfaction with their care, these data have typically been collected under non-pandemic situations, and often among non-interprofessional groups of healthcare staff. When conducting future research on resilience and burnout under pandemic conditions, it would be beneficial to understand how patient satisfaction changes based on the burnout rate of the interprofessional staff overseeing their care. Such information would be beneficial in identifying whether patients feel they are receiving an optimal quality of care even under pandemic conditions from their entire care team, when an entire organization’s operating procedures may be rapidly changing.

Future statistical analyses, including for longitudinal studies, examining the association between resilience and burnout may wish to include demographic variables as covariates. Demographic variables may influence the association between resilience and burnout, particularly in the context of prolonged occupational stress. While subgroup differences in resilience and burnout were not examined in the present study and nor were the impact of specific resilience-building workshops, future research using longitudinal designs and stratified analyses could help identify whether specific professional groups or career stages experience differential benefit from resilience-building interventions, thereby informing targeted workforce strategies. The timing of data collection can also be examined in future longitudinal analyses. Specifically, an examination of how the association between resilience and burnout evolves beyond the acute pandemic phase would support workforce planning and preparedness.

Beyond the scope of healthcare, it would be important to examine if resilience also prevents against burnout in order to help understand how to prepare other employees who could potentially be under significant strain due to pandemic-related pressures (e.g., police officers, teachers, and staff in other workforce sectors that will be impacted by pandemic-related strain).

## 5. Conclusions

Our findings support the previous literature that indicates resilience is tied to the three components of burnout, and demonstrates that this association exists amongst healthcare staff providing patient care under novel pandemic settings. This suggests that resilience remains a relevant construct towards the prevention of burnout amongst clinical and non-clinical patient-facing staff during pandemic conditions. Our data indicated that even when healthcare staff experience EE or DP, they can still achieve a sense of PA from their work. From an organizational and personal standpoint, our results lend credence to the value of regular resilience building and maintenance activities among healthcare staff to reduce burnout and improve wellbeing in the workplace. The organization-wide approach adopted in this study enhances its applicability to system-level wellbeing initiatives, highlighting the need for resilience-building interventions that combine individual resilience-building with structural and organizational supports.

## Figures and Tables

**Table 1:** Demographic Data.

Variable	Overall (*n* = 278)	
	*n*	%
Sex	275	
Male	46	16.7
Female	229	83.3
Age	275	
20–29 years	59	21.4
30–39 years	95	34.5
40–49 years	48	17.5
50–59 years	56	20.4
60–64 years	17	6.2
Undisclosed	3	1.1
Level of Education	275	
Certificate, Diploma, or High School	39	14.2
Master’s	149	54.2
PhD/MD	74	26.9
Undergraduate	13	4.7
Hospital Role	275	
Administrative	12	4.4
Health Disciplines	88	32.0
Nursing	156	56.7
Physicians, Residents, and Fellows	11	4.0
Other	8	2.9

## Data Availability

The data presented in this study are available on request from the corresponding author to protect the privacy of frontline healthcare providers within our organization who took part in this research.

## Abbreviations

The following abbreviations are used in this manuscript:

MBI	Maslach Burnout Inventory
EE	Emotional Exhaustion
DP	Depersonalization
PA	Personal Accomplishment
CD-RISC	Connor-Davidson Resilience Scale
PUIs	Persons under Investigation
